# Supplementary material for: Variation of Structural and Dynamical Flexibility of Myelin Basic Protein in Response to Guanidinium Chloride
Source: Int J Mol Sci. 2022 Jun 23;23(13):6969. doi: 10.3390/ijms23136969 (PMC9266411; doi:10.3390/ijms23136969)
Supplement: Supplementary file 1 [file ijms-23-06969-s001.zip › ijms-1778214-supplementary.pdf]

Supplemental Information

Variation of Structural and Dynamical Flexibility of Myelin Basic Protein in Response to Guanidinium Chloride

Luman Haris[1,2], Ralf Biehl[1], Martin Dulle[1], Aurel Radulescu[3], Olaf Holderer[3], Ingo Hoffmann[4], Andreas M. Stadler*[1,2]

[1] Jülich Centre for Neutron Science (JCNS-1) and Institute of Biological Information Processing (IBI-8), Forschungszentrum Jülich GmbH, 52425 Jülich, Germany

[2] Institute of Physical Chemistry, RWTH Aachen University, Landoltweg 2, 52056 Aachen, Germany

[3] Jülich Centre for Neutron Science (JCNS), outstation at MLZ, Forschungzentrum Jülich GmbH, 85747 Garching, Germany

[4] Institut Laue-Langevin, 71 avenue des Martyrs, CS 20156, 38042 Grenoble Cedex 9, France

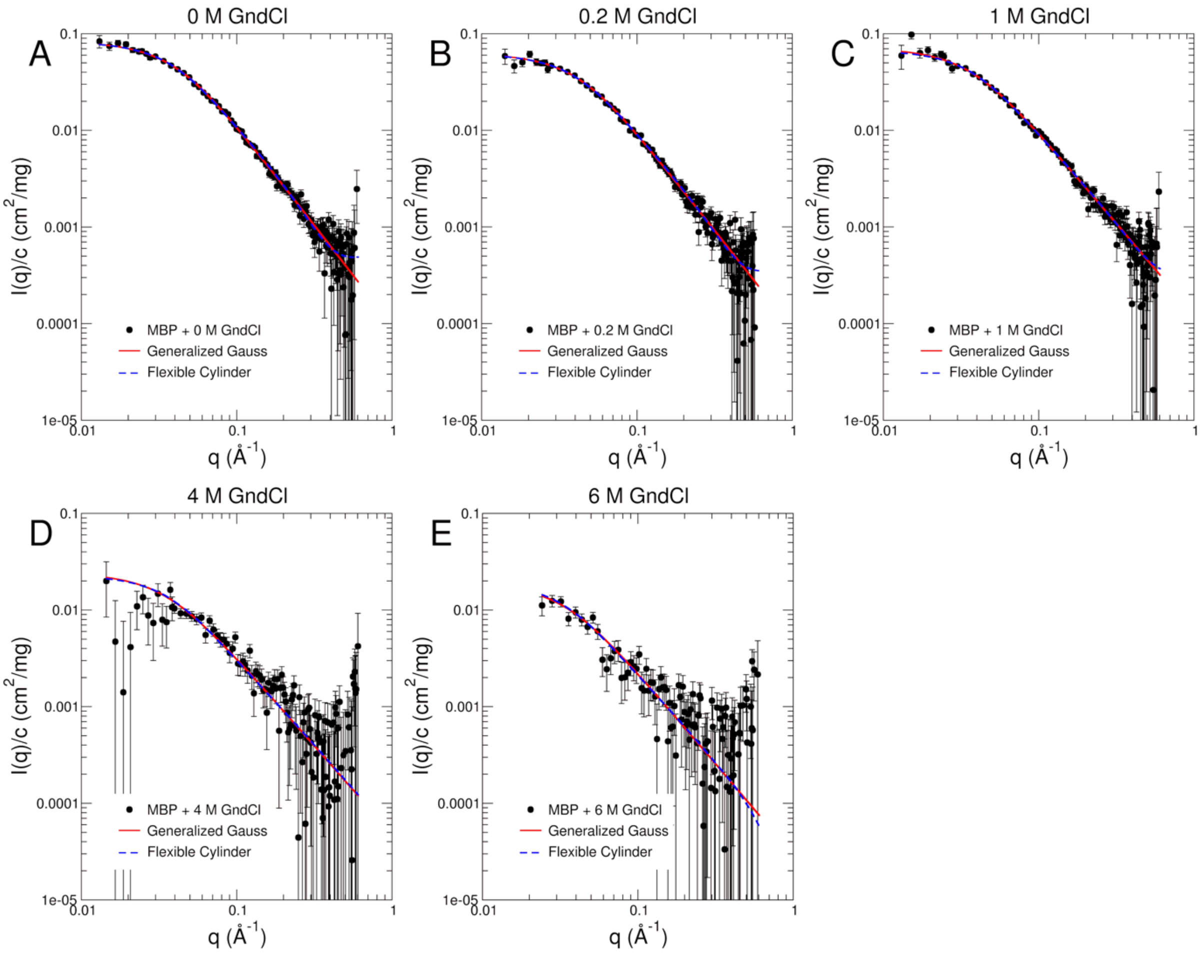

Figure S1 (A-E) Form factors of MBP at 0.5% concentration measured with SAXS at different GndCl concentrations. Experimental SAXS data are fitted with generalized Gauss model (solid red lines) and worm-like flexible cylinder model (dashed blue lines). For clarity only every second experimental data point is shown.

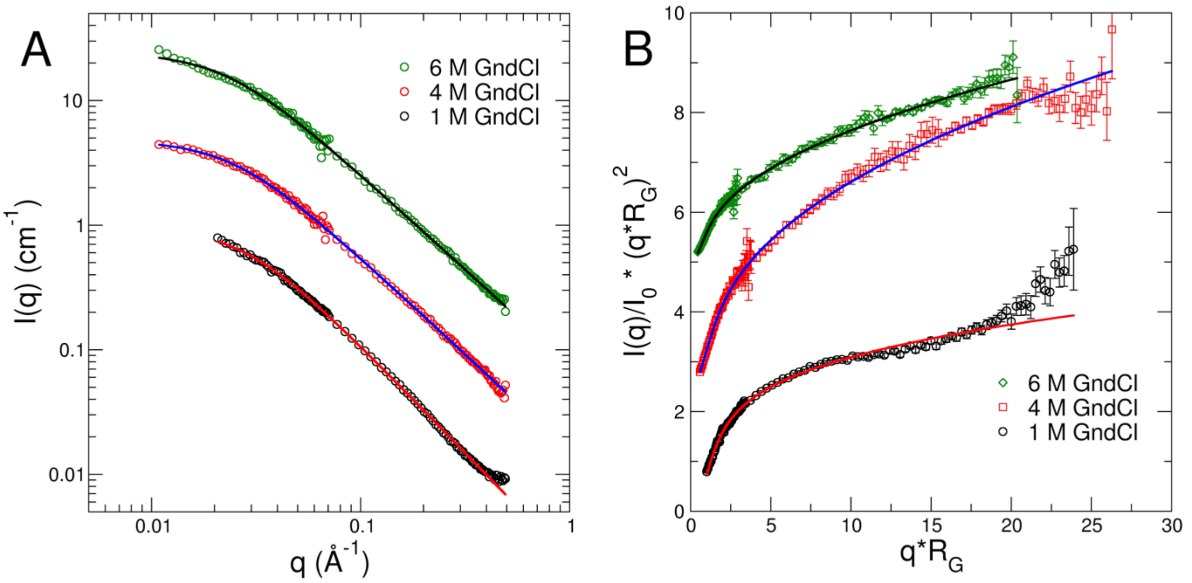

Figure S2 (A) Form factors of MBP at a concentration of 0.5% measured with SANS. (B) Kratky representation of SANS form factors. Solid lines in (A) and (B) are fits to the SANS data with generalized Gauss functions. Experimental data are shifted for clarity.

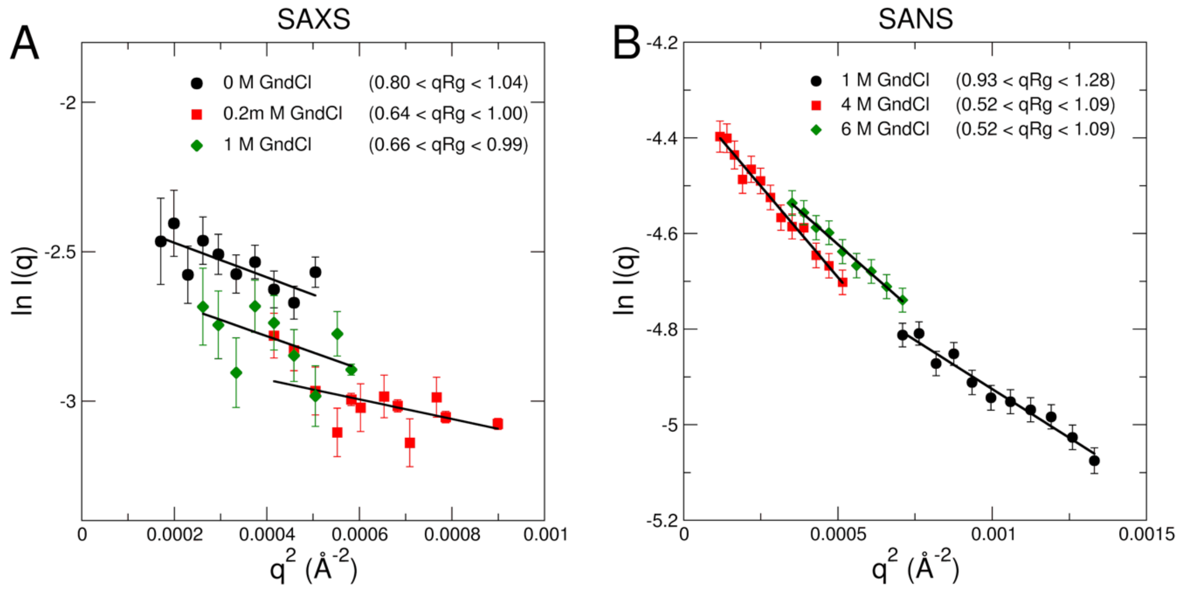

Figure S3 Guinier fits showing (A) SAXS and (B) SANS data at a MBP concentration of 0.5%. The range of the Guinier fits is given in the legend.

Table S1 Input parameters of the worm-like chain model. Among these, only the Kuhn length l_K and the cylinder radius R have been fitted. Scattering length densities (SLD) of D_2O-buffer and MBP have been calculated from the atomic composition, the contour length L was determined from the known MBP sequence length.

c_{GndCl}	**0 M**	**0.2 M**	**1 M**	**4 M**	**6 M**
l_K (Å)	8.1	7.08	8.28	8.78	11.21
R (Å)	6.73	5.93	4.79	3.28	3.37
$SLD_{solvent}$ (10^6 Å^{-2})	9.41	9.45	9.63	10.34	10.85
$SLD_{protein}$ (10^6 Å^{-2})	13.18				
L (Å)	642				

Table S2 Structural parameters of MBP in various GndCl concentrations. Radii of gyration R_G and scaling exponents ν were obtained from SAXS and SANS data. Hydrodynamic radii R_H were measured with DLS.

c_{GndCl}	**0 M**	**0.2 M**	**1 M**	**4 M**	**6 M**
$R_{G,SAXS}$ (nm)	3.72 ± 0.04	3.55 ± 0.05	3.98 ± 0.06	4.20 ± 0.30	4.29 ± 0.14
$R_{G,SANS}$ (nm)	-	-	3.50 ± 0.17	4.45 ± 0.10	4.14 ± 0.19
R_H (nm)	3.80 ± 0.10	4.29 ± 0.03	3.60 ± 0.02	3.41 ± 0.10	3.56 ± 0.30
$R_{G,SAXS}/R_H$	0.98	0.83	1.11	1.23	1.21
ν_{SAXS}	0.481 ± 0.005	0.488 ± 0.007	0.525 ± 0.007	0.550 ± 0.010	0.527 ± 0.020
ν_{SANS}	-	-	0.576 ± 0.003	0.638 ± 0.005	0.650 ± 0.005

Table S3 Structural parameters of coarse-grained EOM models and corresponding population fractions. Coarse-grained models of MBP in different GndCl concentrations have been used for NSE data analysis. Ensemble averaged rotational correlation time $\tau_r = 1/6D_r$

**0 M GndCl**	$R_{G,EOM}$ (Å)	D_{max} (Å)	Population fraction	D_t (Å$^2/ns$)	D_r (ns^{-1}) *10^{-3} / τ_r(ns)
	35.74	99.49	0.07	5.22	3.23
	40.56	136.54	0.14	4.13	2.42
	38.07	112.16	0.07	5.05	2.92
	65.69	202.94	0.14	5.93	5.31
	40.06	111.51	0.07	4.76	2.53
	29.72	100.43	0.21	4.75	2.27
	28.68	82.85	0.29	5.52	3.74
Ensemble	37.87	116.77	1.0	5.14	3.49 / 47 ns
**0.2 M GndCl**	31.06	107.93	0.40	5.53	3.86
	34.46	101.03	0.10	5.2	3.13
	43.70	132.64	0.30	4.94	2.8
	29.39	78.49	0.20	5.55	3.81
Ensemble	34.86	108.77	1.0	5.33	3.46 / 48 ns
**1 M GndCl**	30.40	91.70	0.38	5.28	3.49
	40.81	124.76	0.08	4.6	2.36
	32.59	92.21	0.08	4.91	2.79
	44.82	147.10	0.08	4.44	2.12
	29.58	99.12	0.08	4.4	2.06
	50.93	153.76	0.08	5.16	3.32
	45.90	144.76	0.23	4.57	2.65
Ensemble	38.35	117.52	1.0	4.92	2.87 / 58 ns
**4 M GndCl**	36.67	125.33	0.57	4.23	2.26
	64.84	193.73	0.21	3.59	2.16
	44.11	133.97	0.21	4.0	2.06
Ensemble	44.30	141.84	1.0	4.01	2.18 / 76 ns
**6 M GndCl**	46.69	158.03	0.15	3.08	1.56
	53.49	201.26	0.23	2.86	1.41
	31.84	105.46	0.23	3.62	2.29
	41.74	137.47	0.08	3.25	1.71
	30.64	91.19	0.08	3.59	2.26
	30.85	101.69	0.08	3.68	2.47
	27.61	88.98	0.08	3.24	1.58
	40.75	123.75	0.08	3.91	3.06
Ensemble	40.07	136.87	1.0	3.36	1.97 / 84 ns

Table S4 Results of structure factor analysis of MBP at a concentration of 5% w/v in different GndCl concentrations.

	c_{GndCl}	**0 M**	**0.2 M**	**1 M**	**4 M**	**6 M**
Charged hard spheres	R (nm)	0.4 ± 1.7	0.2 ± 17	-	-	-
	Z (e)	37 ± 26	13 ± 89	-	-	-
	c_{MBP} (mmol)	0.02 ± 148	0.1 ± 8200			
Hard spheres	R (nm)	-	-	2.3 ± 0.6	2.6 ± 0.1	2.8 ± 0.1
	c_{MBP} (mmol)	-	-	0.11 ± 0.08	1.16 ± 0.06	1.32 ± 0.06

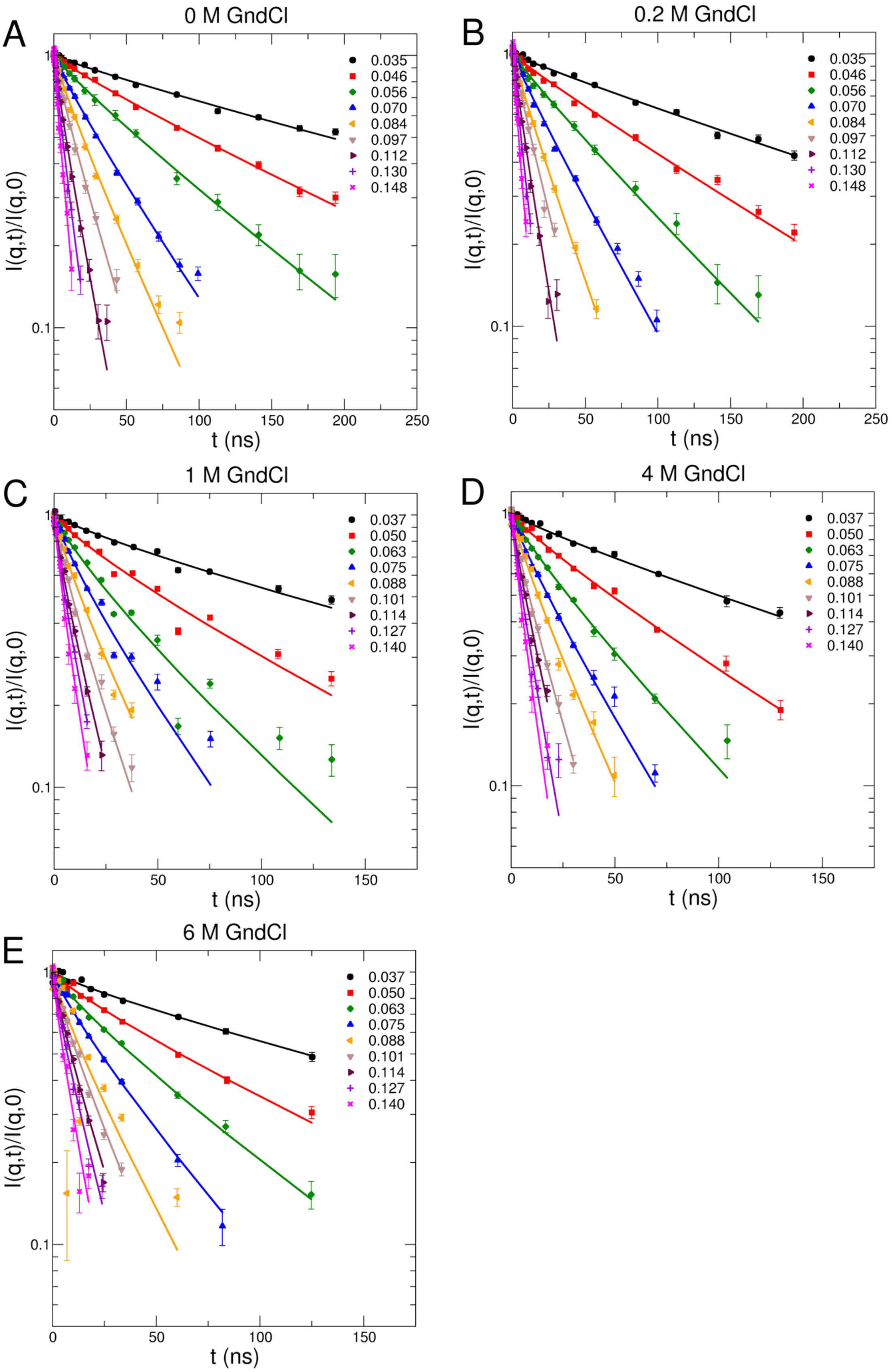

Figure S4 (A-E) NSE spectra of MBP at a concentration of 5% in different GndCl concentrations. Solid lines are fits with stretched exponential functions. Obtained parameters of stretched exponentials are reported in Table S5.

Table S5 Parameters of NSE analysis using stretched exponentials. Average stretching coefficients ⟨β⟩ and the χ^2-values describing the goodness of fit of stretched exponentials to the NSE data set.

c_{GndCl}	**0 M**	**0.2 M**	**1 M**	**4 M**	**6 M**
$\langle\beta\rangle$	0.91 ± 0.009	0.93 ± 0.01	0.84 ± 0.02	0.88 ± 0.02	0.86 ± 0.04
χ^2	1.8	2.9	7.0	2.8	10.7

Table S6 Parameters that were used for the analysis of NSE data. Viscosities η_{buffer} and $\eta_{protein}$ were measured of D_2O-buffers and 5% MBP solutions with viscometry, diffusion coefficients $D_{5\%,DLS}$ were measured with DLS of 5% MBP solutions. Hydrodynamic function H was calculated as the ratio of buffer and protein viscosities. Hydrodynamic functions $H_{NSE\ ZIF}$ and $H_{NSE\ NM}$ were obtained from fits of the ZIF and NM analysis models, respectively, to the NSE data sets. End-to-end distance R_e and bond length l were calculated from SAXS data.

c_{GndCl}	**0 M**	**0.2 M**	**1 M**	**4 M**	**6 M**
η_{buffer} (mPas)	1.26	1.26	1.31	1.47	1.86
$\eta_{protein}$ (mPas)	2.37	2.29	2.63	3.45	4.89
H	0.53	0.55	0.50	0.43	0.38
$H_{NSE\ ZIF}$	0.51	0.53	0.59	0.54	0.54
$H_{NSE\ NM}$	0.55	0.85	0.68	0.50	0.36
$D_{0.5\%,DLS}$ $(Å^2/ns)$	4.5	4	4.58	4.31	3.26
$D_{5\%,DLS}$ $(Å^2/ns)$	1.85	2.64	3.41	4.71	3.66
R_e (nm)	8.97	8.64	10.08	10.33	10.86
l (nm)	2.13	1.99	2.06	2.05	2.22

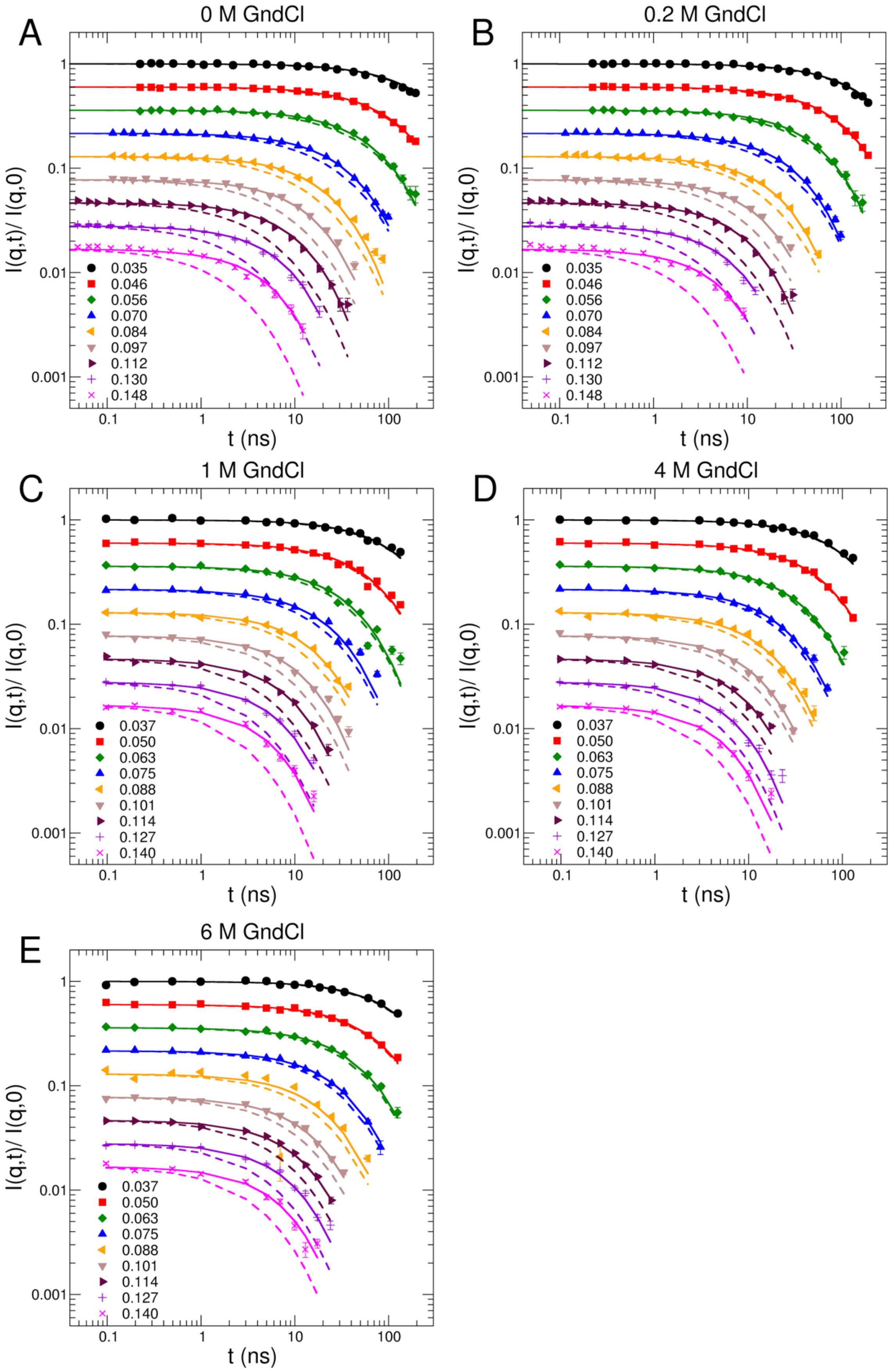

Figure S5 (A-E) NSE spectra of 5% w/v MBP solutions at different GndCl concentrations. Fits were performed with Zimm model (dashed lines) and ZIF model (solid lines).

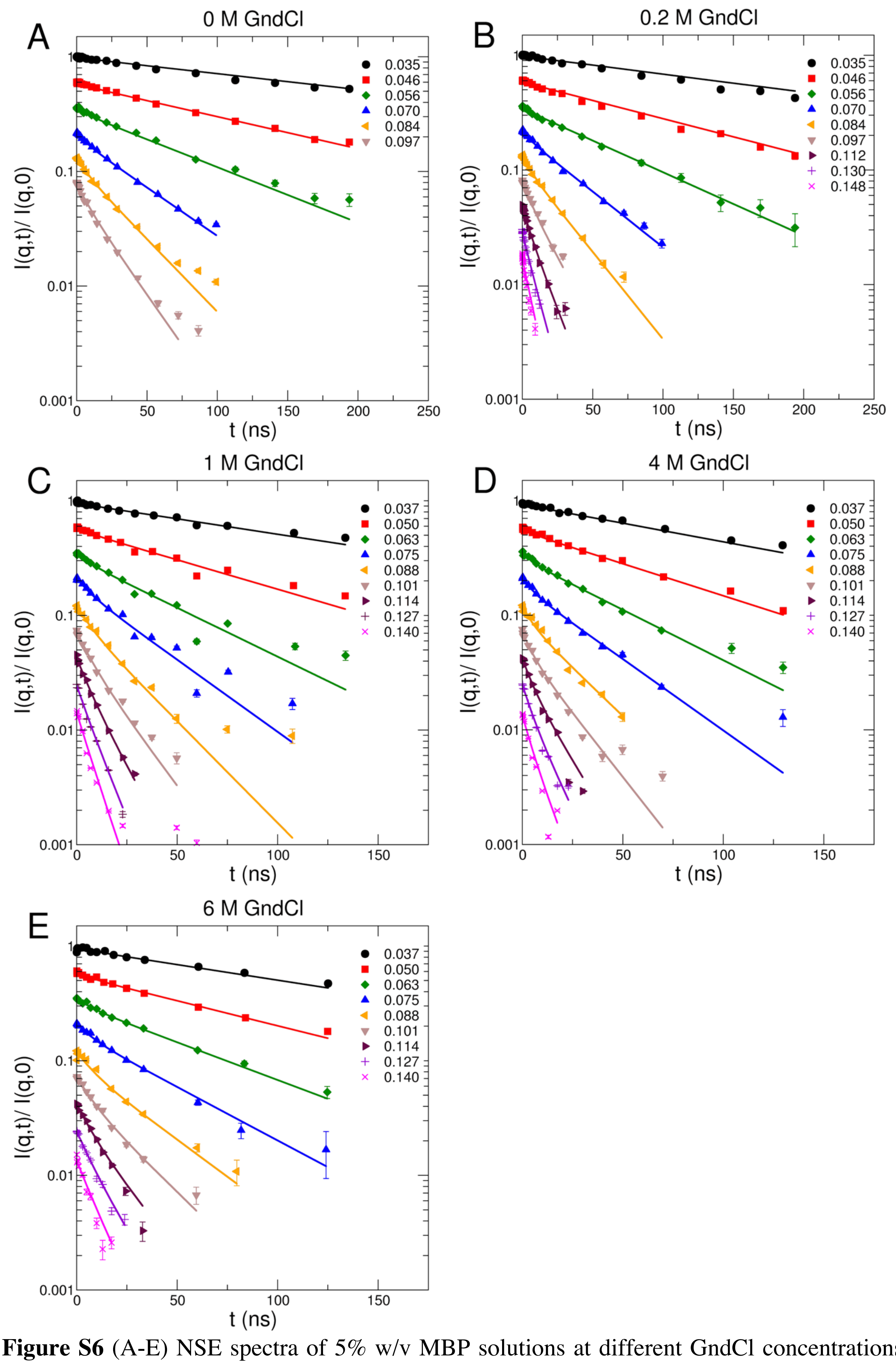

Figure S6 (A-E) NSE spectra of 5% w/v MBP solutions at different GndCl concentrations. NSE spectra fitted with normal mode analysis based on EOM structural ensemble.

Table S7 Goodness of fit χ^2 of the fits of the different models to the NSE data sets. Changing the number of beads *N* does not improve the fits of the ZIF model significantly.

c_{GndCl}	N	0 M	0.2 M	1 M	4 M	6 M	Entire data set
χ^2: Zimm	20	34.6	29.5	22.5	10.4	18.7	23.1
χ^2: Zimm D_{cm} fitted	20	32.7	27.7	31.1	15.6	25.6	26.5
χ^2: ZIF	20	2.4	2.8	8.9	5.6	13.6	6.7
	170	2.4	2.8	9	5.3	13.7	6.6
χ^2: ZIF D_{cm} fitted	20	2.3	2.8	7.5	3.0	10.4	5.2
χ^2: NM H fitted	-	1.9	3.6	7.5	4.0	3.8	4.2

Table S8 Comparison of the first Zimm mode and the relaxation time of internal friction.

	N	0 M GndCl	0.2 M GndCl	1 M GndCl	4 M GndCl	6 M GndCl
$\tau_{p=1}$ (ns)	170	73.3	64.1	102.6	144.4	183.6
τ_i (ns)	170	69.6 ± 5.1	76.6 ± 8.2	49.0 ± 9.0	34.2 ± 4.6	45.0 ± 10.8
